# Association of growth and differentiation factor-15 with coronary artery calcium score and ankle-brachial index in a middle-aged and elderly Caucasian population sample free of manifest cardiovascular disease

**DOI:** 10.1007/s11357-023-00899-y

**Published:** 2023-08-07

**Authors:** Loretta Zsuzsa Kiss, Balázs Bence Nyárády, Éva Pállinger, Árpád Lux, Ádám Levente Jermendy, Csaba Csobay-Novák, Pál Soós, Zsolt Szelid, Orsolya Láng, László Kőhidai, Elek Dinya, Edit Dósa, Béla Merkely, Zsolt Bagyura

**Affiliations:** 1 Heart and Vascular Center, Semmelweis University, 68 Városmajor Street, 1122 Budapest, Hungary; 2 Department of Genetics, Cell- and Immunobiology, Semmelweis University, 4 Nagyvárad Square, 1089 Budapest, Hungary; 3 Department of Cardiology, Cardiovascular Research Institute Maastricht (CARIM), Maastricht University Medical Center, Maastricht, Netherlands; 4 Institute of Digital Health Sciences, Semmelweis University, 15 Ferenc Square, 1094 Budapest, Hungary

**Keywords:** Growth and differentiation factor-15, Coronary artery calcium score, Ankle-brachial index, Atherosclerosis, Cardiovascular risk factors, Aging, Inflammaging

## Abstract

Growth and differentiation factor-15 (GDF-15) is a stress-associated cytokine of the transforming growth factor-β superfamily. The inflammatory and angiogenic effects of GDF-15 in atherosclerosis are controversial, and its correlation with the long asymptomatic phase of the disease is not well understood. Coronary artery calcium score (CACS) and ankle-brachial index (ABI) are sensitive markers of subclinical atherosclerosis. To date, only a few studies have examined the impact of GDF-15 on coronary artery calcification, and the association between GDF-15 and ABI has not been evaluated. Therefore, we aimed to investigate the possible relationship between serum GDF-15 concentrations and CACS and ABI in a Caucasian population sample of middle-aged (35–65 years) and elderly (> 65 years) people. In addition to recording demographic and anthropometric characteristics, atherosclerotic risk factors, and laboratory tests including serum HDL-cholesterol, LDL-cholesterol, hemoglobin A1c (HbA1c), high-sensitivity C-reactive protein, and N-terminal pro-B-type natriuretic peptide (NT-proBNP); GDF-15 level, cardiac computed tomography, and ABI measurements were also performed. A total of 269 asymptomatic individuals (men, *n* = 125; median age, 61.5 [IQR, 12.7] years) formed the basis of this study. Participants were divided into two groups according to their age (middle-aged, *n* = 175 and elderly, *n* = 94). Hypertension and diabetes mellitus were significantly more prevalent and CACS values and HbA1c, NT-proBNP, and GDF-15 levels were significantly higher (all *p* < 0.001) in the elderly group compared to the middle-aged group. Multivariate ridge regression analysis revealed a significant positive association between GDF-15 and CACS (middle-aged group: β = 0.072, *p* = 0.333; elderly group: β = 0.148, *p* = 0.003), and between GDF-15 and ABI (middle-aged group: β = 0.062, *p* = 0.393; elderly group: β = 0.088, *p* = 0.041) only in the elderly group. Our results show that GDF-15 is not only a useful biomarker of inflammation but can also predict early signs of asymptomatic atherosclerosis, especially in elderly people with chronic systemic inflammation associated with aging (inflammaging).

## Introduction

Growth and differentiation factor-15 (GDF-15) is a contradictory member of the transforming growth factor-β superfamily [1]. It is a stress-sensitive cytokine [2]; the production of GDF-15 by macrophages, endothelial cells, vascular smooth muscle cells, adipocytes, and some other cells is accelerated under pathological conditions (proinflammatory state, vascular injury, pressure overload, etc.) [3]. Depending on the cellular context, the microenvironment, or the stage of the disease, GDF-15 may have opposing effects (e.g., pro- and anti-inflammatory, pro- and anti-apoptotic, and pro- and anti-angiogenic) [4].

Its normal range is 200–1200 pg/mL; serum levels increase with age [5]. Higher than normal serum concentrations of GDF-15 have been described in the following non-atherosclerotic conditions or diseases: neurocognitive impairment, pulmonary hypertension, hypertension, atrial fibrillation, heart failure, non-alcoholic fatty liver disease, end-stage kidney disease, cachexia, obesity, diabetes mellitus, metabolic syndrome, chronic inflammatory diseases, and malignancies [5].

The exact relationship between GDF-15 and atherosclerosis is not fully understood. In mouse models, GDF-15 has both deleterious and protective effects. In the early phases of atherosclerosis, GDF-15 promotes chemotaxis of macrophages to plaques [6]. In advanced atherosclerosis, the available data on GDF-15 are contradictory; overexpression of GDF-15 reduces plaque size, while lack of GDF-15 improves plaque stability by impairing macrophage migration and enhancing collagen deposition [7, 8].

In humans, serum levels of GDF-15 have been shown to be elevated in both subclinical and clinical forms of atherosclerosis [9, 10]. Two of the most important markers of subclinical atherosclerosis are the coronary artery calcium score (CACS; also known as Agatston score) and the carotid intima-media thickness [11, 12]. Besides these two, some studies also mention the ankle-brachial index (ABI) as a general parameter of subclinical atherosclerotic burden [13, 14]. To the best of our knowledge, the impact of GDF-15 on coronary artery calcification has only been examined in a few studies [15–18], and the association between GDF-15 and ABI has not been assessed. Of the studies looking at the link between GDF-15 and CACS, only one was population-based; this population was multiethnic and included only participants under 65 years of age [15].

Growth and differentiation factor-15 predisposes not only to the manifestation of the atherosclerotic process but also to its progression [19]. Among the atherosclerotic manifestations, acute coronary syndrome (ACS), ischemic stroke, and peripheral artery disease (PAD) should be highlighted. The cytokine GDF-15, independently of classical cardiovascular risk factors, clinical predictors, number of affected vessels, and other biomarkers (e.g., cardiac troponin I, N-terminal pro-B-type natriuretic peptide [NT-proBNP], high-sensitivity C-reactive protein [hs-CRP], and creatinine clearance), provides prognostic information about ACS, which is a consequence of erosion or rupture of coronary plaques and can lead to recurrent myocardial infarction (MI) and/or death [20]. Circulating levels of GDF-15 in PAD patients at risk of major amputation and/or death are also generally well above the normal range [21].

The aim of our study was to investigate the relationship between serum GDF-15 concentrations and CACS and ABI in a Caucasian population sample consisting of both middle-aged (35–65 years) and elderly (> 65 years) people.

## Methods

The present study was part of the Budakalász Health Survey, a cross-sectional voluntary (cardio)vascular screening program targeting the adult (> 20 years) population of the city of Budakalász in 2011–2013 [22]. The study was approved by the Scientific and Ethics Committee of the Medical Research Council (approval number 8224–0/2011/ECU [265/PI/11]). All procedures were conducted in accordance with the ethical standards of the relevant (institutional and national) committee on human experiments and the 1975 Declaration of Helsinki, revised in 2000 [23]. Written informed consent was requested from all participants.

In addition to determining demographic (age and sex) and anthropometric characteristics (weight and height), a medical history was taken, focusing on risk factors for atherosclerosis (smoking, hypertension, and diabetes mellitus) and the presence and management of (cardio)vascular diseases (transient ischemic attack [TIA], stroke, angina pectoris, MI, cardiomyopathy, heart failure, and PAD), and laboratory tests were performed. The routine laboratory parameters were high-density lipoprotein cholesterol (HDL-C) and low-density lipoprotein cholesterol (LDL-C), while the other laboratory parameters were hemoglobin A1c (HbA1c), hs-CRP, NT-proBNP, and GDF-15.

As described in our former work in detail [24], anthropometric characteristics were measured on standing participants wearing lightweight, shoeless, indoor clothing. Body mass index (BMI) was calculated using the Quetelet formula. A participant was defined as an active smoker if he/she smoked at least one cigarette a day. A diagnosis of hypertension and diabetes mellitus was considered to exist if there was evidence of it in the patient's previous medical records and/or if the patient had been treated for it. Laboratory tests were carried out in our central laboratory under strict quality control. The concentration of lipid fractions was evaluated by enzymatic colorimetric assay (Beckman Coulter DxC 700 AU, Beckman Coulter Inc., Brea, CA, USA). The levels of HbA1c and hs-CRP were measured by immunoturbidimetric method (Beckman Coulter DxC 700 AU), while NT-proBNP levels were determined by electrochemiluminescence technique (Roche/Hitachi Cobas e411, F. Hoffmann-La Roche Ltd., Basel, Switzerland). Plasma GDF-15 levels were quantified by bead-based flow cytometry (AimPlex assay technology, AimPlex Biosciences Inc., Pomona, CA, USA) according to the manufacturer's instructions.

Men over 35 and women over 40 were given the opportunity to have a cardiac computed tomography (CT) scan to assess CACS (also known as Agatston score). For those who consented, a prospectively electrocardiogram-triggered low dose (≤ 0.5 mSv) CT scan (Brilliance iCT, Philips Healthcare, Best, The Netherlands) with a narrow field-of-view was done. Coronary plaque delineation and quantitative analysis were conducted on axial plane images with commercially available semi-automated software (Calcium scoring, Heartbeat-CS, Philips Healthcare). Coronary plaques were defined as circumscribed lesions ≥ 1 mm^2^ in area and > 130 Hounsfield units in density. The plaques selected by the software were checked by a specialist and manually modified if necessary. The software then determined the Agatston score based on the total area of calcium deposits and calcium density. The methods applied were the same as in reference 25. [25]

The ABI was assessed after participants had rested in a supine position for at least 5 min and then had their systolic blood pressure measured on both arms and legs with an appropriately sized cuff. In both legs, a 5 MHz continuous wave Doppler ultrasound probe was used to detect the flow of the dorsalis pedis artery and the posterior tibial artery. The leg-specific ABI was calculated by dividing the higher of the dorsalis pedis artery and posterior tibial artery systolic blood pressures by the higher of the left and right brachial systolic blood pressures. For the analyses, the lower of the two leg-specific ABIs was chosen. The ABI was considered abnormal if it was above 1.4 or below 0.9.

### Statistical analysis

For statistics, Excel and SPSS for Windows version 25.0 (IBM, Armonk, NY, USA) were used. Due to the distribution of the data, continuous variables were given as median and interquartile range (IQR) and compared with Mann–Whitney *U*-test. Categorical variables were expressed as numbers and percentages and compared with the Chi-square test. The relationship between GDF-15 and CACS was examined using Spearman's correlation, while the relationship between GDF-15 and ABI was assessed using logistic regression. Ridge regression analysis adjusted for sex, age, and atherosclerotic risk factors was performed to test the association between GDF-15 and CACS and ABI in a multivariate model. All analyses were two-tailed and *p* < 0.05 was considered significant.

## Results

**Fig. 1:**
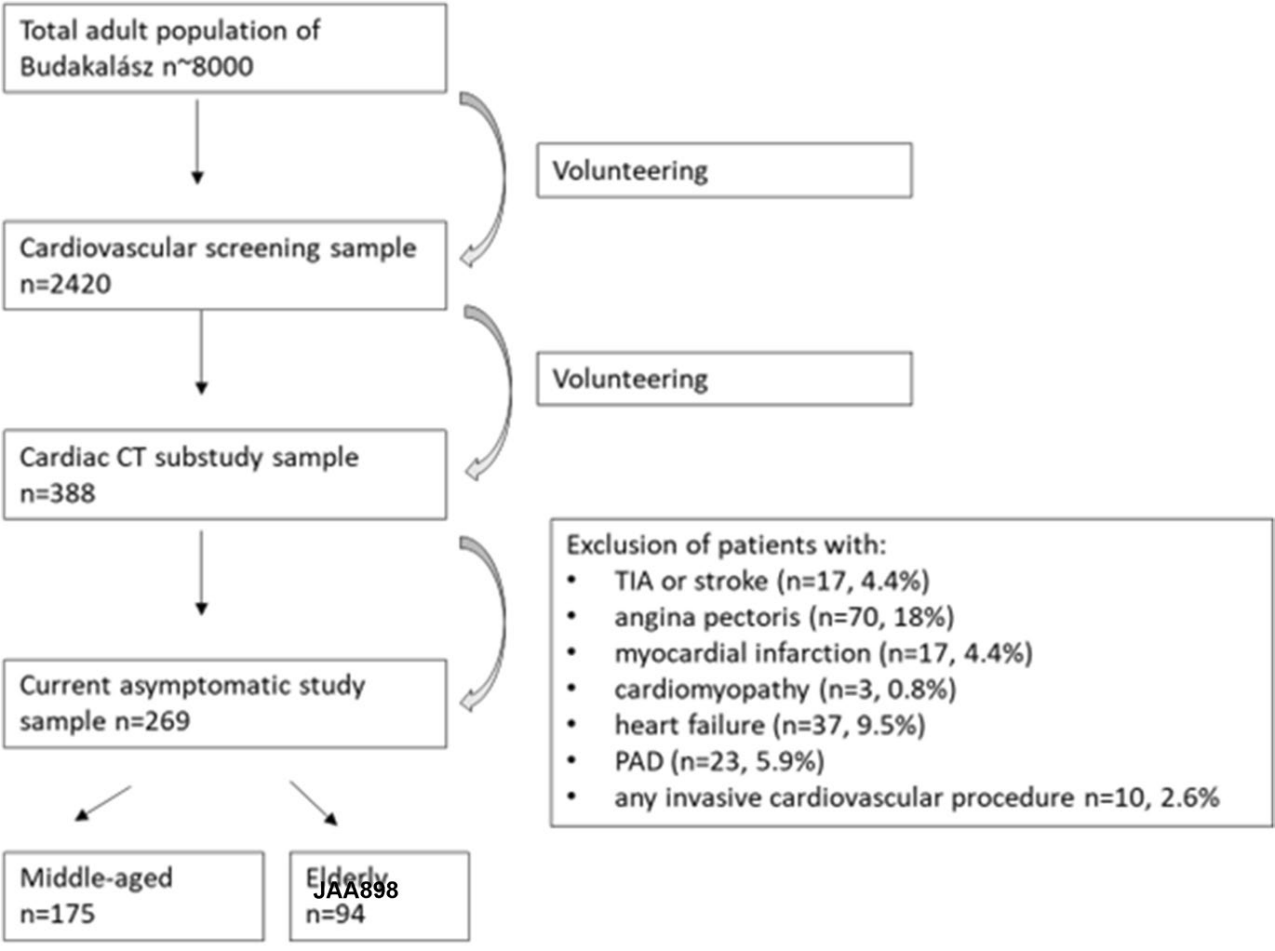
Study flowchart

The number of participants for whom all data were available was 388. Those with a history of TIA or stroke (*n* = 17 [4.4%]), angina pectoris (*n* = 70 [18%]), MI (*n* = 17 [4.4%]), cardiomyopathy (*n* = 3 [0.8%]), heart failure (*n* = 37 [9.5%]), and/or symptomatic PAD (*n* = 23 [5.9%]), and who underwent any invasive procedure as a result (*n* = 10 [2.6%]), were excluded from further analysis. Thus, a total of 269 asymptomatic participants formed the basis of our study (Fig. 1).

All participants were Caucasian, ranged in age from 35 to 85 years (median age, 61.5 years [IQR, 12.7 years]), and 46.5% were male. Participants were divided into two groups according to their age (middle-aged and elderly). One hundred and seventy-five participants (65.1%) belonged to the middle-aged group (35–65 years) and 94 participants (34.9%) to the elderly group (> 65 years). Participants' demographics, cardiovascular risk factors, and HDL-C, LDL-C, and HbA1c levels are presented in Table 1. Twenty-nine participants (10.8%) were active smokers and 139 participants (51.7%) and 28 participants (10.4%) had known hypertension and diabetes mellitus, respectively. Participants had a median BMI of 27.7 kg/m^2^ (IQR, 6.6 kg/m^2^), a median HbA1c value of 5.7% (IQR, 0.6%), a median HDL-C level of 1.4 mmol/L (IQR, 0.6 mmol/L), and a median LDL-C level of 3.5 mmol/L (IQR, 1.3 mmol/L). Hypertension and diabetes mellitus were significantly more common (both *p* < 0.001) and HbA1c values were significantly higher (*p* < 0.001) in the elderly group than in the middle-aged group. (Table 1).

**Table 1:** Participants' demographics, cardiovascular risk factors, and levels of high-density lipoprotein cholesterol, low-density lipoprotein cholesterol, and hemoglobin A1c

Demographics, cardiovascular risk factors, lab parameters	Whole group (*n* = 269)	Middle-aged group (*n* = 175)	Elderly group (*n* = 94)	*p*-value
Age (years), median (IQR)	61.5 (12.7)	57.5 (12.7)	69.1 (5.1)	< 0.001
Male, n (%)	125 (46.5)	85 (48.6)	40 (42.6)	NS
Active smoker, n (%)	29 (10.8)	23 (13.1)	6 (6.4)	NS
Hypertension, n (%)	139 (51.7)	79 (45.1)	60 (63.8)	< 0.001
Diabetes mellitus, n (%)	28 (10.4)	7 (4)	21 (22.3)	< 0.001
BMI (kg/m^2^), median (IQR)	27.7 (6.6)	27.7 (7)	27.4 (6.8)	NS
HDL-C (mmol/L), median (IQR)	1.4 (0.6)	1.4 (0.6)	1.4 (0.6)	NS
LDL-C (mmol/L), median (IQR)	3.5 (1.3)	3.5 (1.3)	3.5 (1.4)	NS
HbA1c (%), median (IQR)	5.7 (0.6)	5.6 (0.5)	5.9 (0.8)	< 0.001

*BMI*, Body mass index; *HbA1c*, hemoglobin A1c; *HDL-C*, high-density lipoprotein cholesterol; *IQR*, interquartile range; *LDL-C*, low-density lipoprotein cholesterol; *NS*, non-significant

For the whole group, the median CACS was 15 (IQR, 100), the median GDF-15 level was 217 pg/mL (IQR, 204 pg/mL), the median NT-proBNP level was 70.4 pg/mL (IQR, 99.5 pg/mL), and the median hs-CRP level was 1.6 mg/L (IQR, 2.6 mg/L). Abnormal ABI was measured in 33 participants (12.3%). With the exception of abnormal ABI and hs-CRP levels, biomarker values/levels were significantly higher (all *p* < 0.001) in the elderly group than in the middle-aged group. (Table 2).

**Table 2:** Participants' subclinical atherosclerotic parameters and levels of growth and differentiation factor-15, N-terminal pro-B-type natriuretic peptide, and high-sensitivity C-reactive protein

Biomarkers	Whole group (*n* = 269)	Middle-aged group (*n* = 175)	Elderly group (*n* = 94)	*p*-value
CACS, median (IQR)	15 (100)	3.3 (48)	67 (321)	< 0.001
Abnormal ABI, n (%)	33 (12.3)	22 (12.6)	11 (11.7)	NS
GDF-15 (pg/mL), median (IQR)	217 (204)	174 (162)	291 (227)	< 0.001
NT-proBNP (pg/mL), median (IQR)	70.4 (99.5)	55.4 (66)	109 (110)	< 0.001
hs-CRP (mg/L), median (IQR)	1.6 (2.6)	1.6 (3)	1.7 (2.2)	NS

*ABI*, Ankle-brachial index; *CACS*, coronary artery calcium score; *GDF-15*, growth and differentiation factor-15; *hs-CRP*, high-sensitivity C-reactive protein; *IQR*, interquartile range; *NS*, non-significant; *NT-proBNP*, N-terminal pro-B-type natriuretic peptide

In the whole group, GDF-15 showed a significant positive association with both CACS (correlation: r = 0.339, *p* < 0.001) and ABI (logistic regression: OR = 1.001, *p* = 0.027). Multivariate ridge regression analysis revealed a significant positive correlation between GDF-15 and CACS (middle-aged group: β = 0.072, *p* = 0.333; elderly group: β = 0.148, *p* = 0.003), and between GDF-15 and ABI (middle-aged group: β = 0.062, *p* = 0.393; elderly group: β = 0.088, *p* = 0.041) only for older participants.

## Discussion

The current understanding is that inflammation plays an important role in the progression of atherosclerosis and could also trigger vascular calcification [26]. Previously, we have reported our results on the association of neutrophil-to-lymphocyte ratio, a marker of inflammation and coronary calcification [27].

In this study, GDF-15 was found to be a significant and independent factor affecting coronary artery calcification in individuals over 65 years of age without a history of cerebrovascular, cardiovascular, or peripheral artery disease.

The association of GDF-15 and coronary artery calcification has already been evaluated in five other studies. The most comprehensive of these studies was the Dallas Heart Study (DHS), which included 3219 participants [15]. This population-based study, like ours, reported a significant positive correlation between serum levels of GDF-15 and CACS. However, there are important differences between our study and the DHS in terms of population composition, age distribution of participants, and use of CACS. Our population was homogeneous, but about 50% of the DHS participants were African-American. While the DHS only included those under 65, our results also covered participants over 65. The analysis of coronary artery calcification in both studies was based on the Agatston scoring system, but while we used Agatston scores as continuous variables, they created categories with cut-off values of 10 and 100. Similarly, Hacıoğlu et al. [28] assessed coronary artery calcification in 86 volunteers (aged 25–85 years) and divided the patients into two groups, with a cut-off of 0 Agatston score.

The other three studies investigating the relationship between GDF-15 and coronary artery calcification all involved specific patient populations: patients with chronic obstructive pulmonary disease (COPD)[16], end-stage renal disease[17], and psoriasis [18]. Martinez et al.[16] noted in a cross-sectional study of 694 COPD patients in the COPDGene cohort that GDF-15 was independently related to coronary artery calcification in smokers without known cardiovascular disease. Laučytė-Cibulskienė et al.[17] observed that in 151 male patients with end-stage renal disease, serum levels of GFD-15 were significantly associated with CACS, and those with CACS above 400 had significantly higher GDF-15 concentrations than those with CACS below 400. Kaiser et al.[18] performed a large‐scale plasma proteomic analysis in 85 patients with psoriasis. They showed that GDF-15 was positively linked to CACS in patients without cardiovascular disease and receiving statin therapy.

In our study, elevated levels of serum GDF-15 have been shown to be positively associated with abnormal ABI in both middle-aged and elderly groups. To the best of our knowledge, the association between GDF-15 and ABI, a subclinical marker of PAD, has not been investigated at all, and the impact of GDF-15 on manifest PAD has been assessed in only two studies. In a prospective observational study, Jönelid et al.[29] found a positive correlation between GDF-15 concentrations and PAD in patients who had recently had a heart attack. Like us, they considered ABI values of < 0.9 and > 1.4 to be abnormal. Dakhe et al.[30] studied a cohort of 267 men aged 65 years with abdominal aortic aneurysms and reported that high levels of GDF-15 increased the risk of PAD, defined as an ABI < 0.9.

Aging is a major risk factor for almost all chronic diseases. Older age is often associated with dysregulation of the immune system and a low-grade chronic inflammatory state, so-called inflammaging [31]. As mentioned earlier, GDF-15 plays a key role in the inflammatory immune processes that are present in chronic diseases [32]. Tanaka et al.[33] examined the plasma proteome and revealed that of the myriad immune parameters, GDF-15 showed the strongest linear correlation with age. This result has since been confirmed by several other publications. It therefore appears that GDF-15 is a potential biomarker of inflammaging.[34] Higher levels of GDF-15 can mark low-grade chronic systemic inflammation, which may be part of the aging process, so it is possible that the balance in the elderly is tipped in the proinflammatory direction. Moreover, their cardiovascular system may be more vulnerable to both exogenous and endogenous stressors including the effects of GDF-15.[35] In the United States and Western European countries, average life expectancy has increased significantly in recent decades [36]. In our country, adults aged 65 and over make up ~ 20% of the population and this number is expected to rise further [37]. This, therefore, makes the identification of aging-related biomarkers particularly important. As measurement of GDF-15 is performed by a common method, its use in everyday clinical practice would be feasible.

In conclusion, it seems that GDF-15 is not only a useful biomarker of inflammation but can also predict early signs of asymptomatic atherosclerosis, especially in elderly people with chronic systemic inflammation associated with aging (inflammaging).

### Limitations

Our study has certain limitations. The composition of our study sample reflects the fact that it was based on a voluntary screening, thus higher-risk but more health-conscious individuals may have had a greater propensity to participate than healthy ones, so our sample may not be representative.

## Data Availability

Data are available upon reasonable request from the authors.
